# Non‐Small Cell Lung Carcinoma With Bilayered Architecture and *TP53* Alteration: A Diagnostic Pitfall and a Potential Distinct Entity

**DOI:** 10.1111/1759-7714.70411

**Published:** 2026-10-29

**Authors:** Yao Liu, Biao Zhang, Xiaobin Cui, Fanqing Meng

**Affiliations:** ^1^ Department of Pathology Nanjing Drum Tower Hospital, the Affiliated Hospital of Nanjing University Medical School Nanjing Jiangsu Province China

**Keywords:** bilayered carcinoma, bronchiolar adenoma, *TP53* mutation

## Abstract

The presence of a continuous basal cell layer is generally regarded as a defining feature of bronchiolar adenoma/ciliated muconodular papillary tumor (BA/CMPT), and preservation of this bilayered architecture is commonly used as a surrogate marker of benign behavior. We report a 1.1‐cm pulmonary nodule detected in a 75‐year‐old never‐smoker that exhibited marked high‐grade cytologic atypia in both luminal and basal cells. Immunohistochemical analysis demonstrated diffuse strong P53 expression and an increased proliferative index (Ki‐67 approximately 10%). Targeted next‐generation sequencing revealed a pathogenic *TP53* mutation (p.A161T). Despite the intact basal layer, the diffuse high‐grade cytology and shared strong P53 expression in both compartments indicate that this lesion represents a fully malignant carcinoma rather than a benign BA/CMPT. In practice, it can only be classified as non‐small cell lung cancer (NSCLC), NOS, with a distinctive bilayered phenotype. This case emphasizes that a preserved basal cell layer does not always equate to benignity, and it highlights a rare tumor type that may mimic BA/CMPT, carrying a risk of underdiagnosis. Integrating cytology, immunophenotype, and molecular findings is crucial for correct classification.

## Graphical abstract

An intact basal cell layer does not always indicate benignity; high‐grade cytology, *TP53* mutation, and increased proliferation support classification as NSCLC, NOS with a distinctive bilayered phenotype.

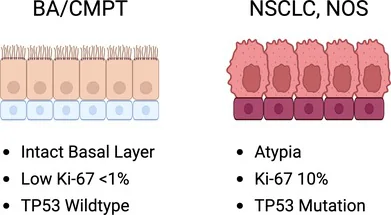

## 1 Introduction

Bronchiolar adenoma/ciliated muconodular papillary tumor (BA/CMPT) is a rare distal airway tumor, generally regarded as indolent and defined by a characteristic bilayered architecture composed of basal and luminal epithelial cells. In routine practice, an intact basal cell layer is often interpreted as evidence against a malignant process. However, the biological significance of high‐grade cytologic atypia arising within a lesion that retains this bilayered pattern remains poorly understood.

Here, we report an unusual pulmonary carcinoma that maintains a continuous bilayered architecture throughout the entire 1.1‐cm nodule, yet both layers exhibit high‐grade atypia and harbor a *TP53* mutation. This case challenges the prevailing assumption that preservation of a continuous basal cell layer reliably excludes malignancy, and it draws attention to a potential diagnostic pitfall.

## 2 Clinical Summary

A 75‐year‐old male, non‐smoker, was found to have an incidental peripheral lung nodule on routine computed tomography. The 11 × 10 mm nodule in the posterior segment of the left upper lobe was partly solid, with lobulated margins, spiculations, and an air bronchogram, features concerning for a high‐risk lesion (Figure 1A). The patient underwent a left upper lobe posterior segmentectomy.

**FIGURE 1:**
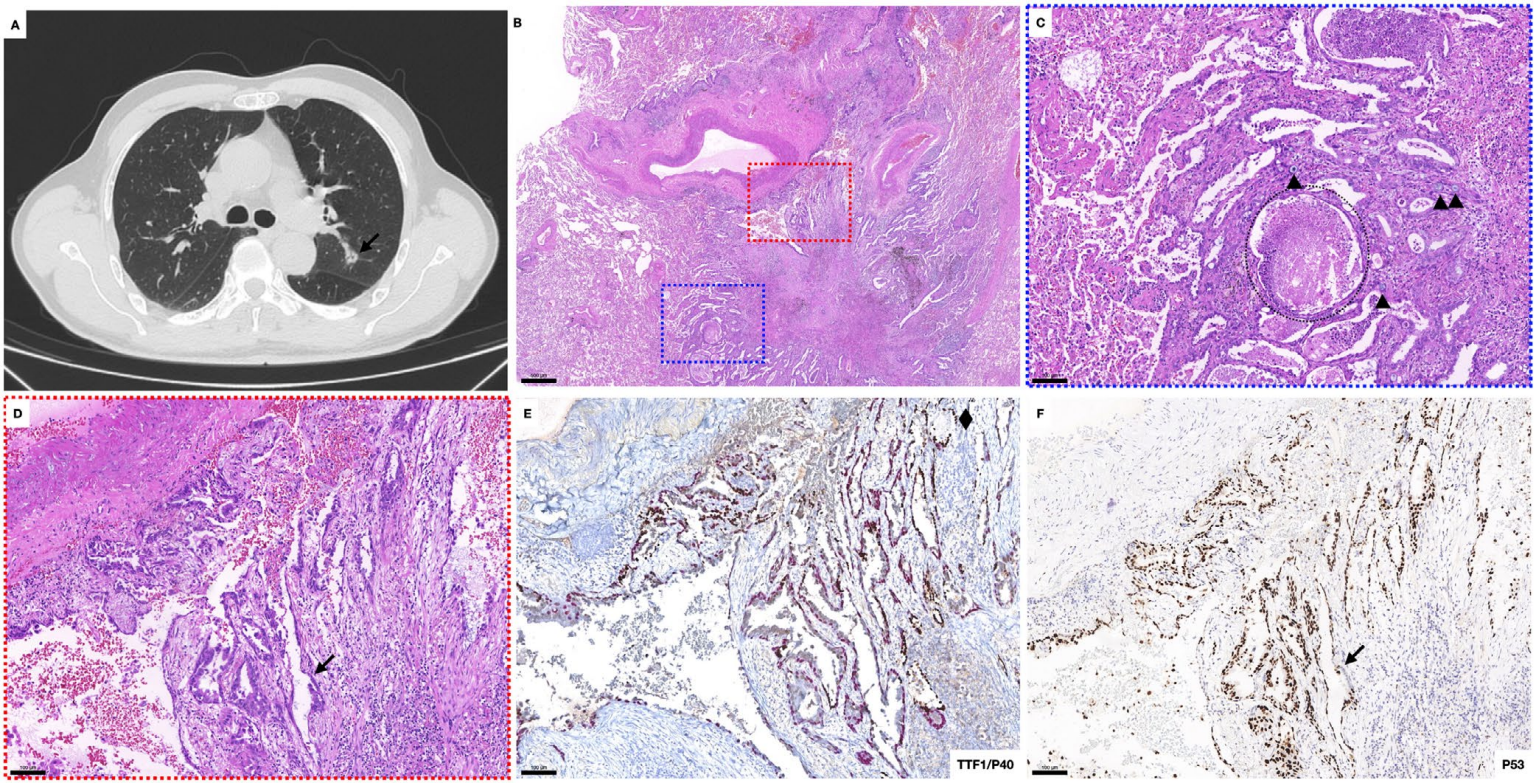
Radiologic, histologic, and immunohistochemical findings. (A) Chest CT image showing a partly solid pulmonary nodule with lobulated margins and spiculation. (B) Low‐power H&E view of the lesion adjacent to a small pulmonary artery. (C) High‐power H&E reveals marked cytologic atypia in luminal cells, with focal intraluminal necrosis (circle) and aberrant mucin production (triangle). (D) High‐power H&E showing the preserved bilayered architecture (arrow indicates tumor‐normal interface). (E) Dual immunohistochemical stain (TTF‐1/P40) demonstrating a continuous basal cell layer (P40+, red). Tumor cells show relatively weak TTF‐1 expression (brown), whereas adjacent alveolar epithelium shows strong TTF‐1 positivity (diamond). (F) P53 immunohistochemistry showing diffuse strong nuclear positivity in both tumor cell layers (arrow marks sharp transition from normal epithelium). Scale bars: Black bars = 100 μm (C–F) and 500 μm (B).

## 3 Pathological Findings

Histologic examination demonstrated a lesion with a well‐preserved bilayered architecture throughout the entire nodule (Figure 1B–D). The basal cell layer was continuous, as demonstrated by a TTF‐1/P40 dual immunohistochemical stain (Figure 1E). In contrast to the bland cytology expected in BA/CMPT, the luminal epithelial cells exhibited marked nuclear pleomorphism, hyperchromasia, focal intraluminal necrosis, and aberrant mucin production, consistent with high‐grade cytologic atypia (Figure 1C,D). Notably, the basal cells also displayed atypical features. Both layers exhibited diffuse, strong nuclear positivity for P53 (Figure 1F), and the Ki‐67 labeling index was elevated at 10%. Two independent pathologists initially favored adenocarcinoma; however, the intact basal layer argued against stromal invasion. Targeted next‐generation sequencing with the SimcereDx 91‐gene lung cancer panel (Supplementary Table 1) revealed a pathogenic *TP53* mutation and a concurrent *IDH2* mutation at low allele frequency (0.029) (Table 1). Molecular separation of the two cell layers for sequencing was technically unfeasible due to their intimate intermingling. Given the high‐grade cytology and shared *TP53* mutation, the lesion was ultimately diagnosed as non‐small cell lung cancer (NSCLC), NOS, with a distinctive bilayered architecture.

**TABLE 1:** Somatic mutations identified by next‐generation sequencing.

Gene	Genomic change	Protein change	Allele frequency	Pathogenicity
*TP53*	chr17:7578449 C>T	p.Ala161Thr	11.8%	Pathogenic
IDH2	chr15:90631934 C>T	p.Arg140Gln	2.9%	Oncogenic

## 4 Discussion

This case represents a rare carcinoma characterized by a deceptive bilayered architecture, high‐grade cytologic atypia and diffuse aberrant P53 expression—features that would be incompatible with a diagnosis of BA/CMPT. Classic BA/CMPT typically displays bland cytology, low proliferative activity, and recurrent alterations involving *BRAF*, *KRAS*, or less frequently *EGFR* [1], and the absence of nuclear atypia is considered an essential diagnostic criterion. Reports of malignant transformation are exceedingly rare [2, 3], and in those instances, a well‐demarcated benign BA/CMPT component is still identifiable adjacent to the carcinoma. In the present lesion, no such benign precursor area is present; even the most innocuous‐appearing foci harbor the same aberrant p53 expression and *TP53* mutation as the overtly atypical regions.

In pulmonary adenocarcinoma, *TP53* alterations are generally associated with tumor progression and genomic instability [4]. The presence of a pathogenic *TP53* alteration accompanied by diffuse p53 overexpression in both epithelial layers suggests that the genetic alteration arose in a shared progenitor population, giving rise to a neoplasm that preserves a structured bilayer yet is fully malignant. This phenomenon is conceptually reminiscent of urothelial carcinoma in situ, where full‐thickness atypia exists without invasion. An important differential diagnosis is conventional invasive adenocarcinoma colonizing the luminal surface of peribronchiolar metaplasia (cancerization) [5]. We carefully considered this possibility but found no supporting evidence. The entire 1.1‐cm nodule uniformly displays a bilayered structure, with no area exhibiting the single‐layered infiltrative growth of typical adenocarcinoma, nor any separate invasive component from which cancerization could originate. Moreover, the presence of strong P53 expression in both compartments confirms that the bilayered organization itself is neoplastic. Although *IDH2* mutations are uncommon in lung adenocarcinoma and may be subclonal events [6], they have not been reported in BA/CMPT sequencing series [1] and are well recognized in several malignant neoplasms [7]. In the present case, the very low allele frequency (0.029) supports a possible passenger event; nevertheless, its detection provides additional molecular context favoring carcinoma rather than BA/CMPT.

From a clinical standpoint, the optimal surgical approach remains undefined. This patient underwent anatomical segmentectomy and remains recurrence‐free; however, whether wedge resection would have been sufficient is uncertain. During intraoperative consultation, the bilayered architecture is difficult to appreciate, and such a lesion would almost certainly be interpreted as adenocarcinoma based on cytology alone. Thus, definitive diagnosis relies on formalin fixed paraffin‐embedded (FFPE) sections with immunohistochemistry. Until more cases are recognized, we recommend that bilayered lesions with high‐grade cytology be managed as malignant, with complete excision and careful surveillance. Future studies utilizing layer‐specific genomic profiling are needed to further characterize this rare entity.

## 5 Conclusion

We report a pulmonary carcinoma with a preserved bilayered architecture but harboring diffuse high‐grade cytologic atypia and a pathogenic *TP53* alteration. It does not meet the criteria for BA/CMPT. Because there is currently no appropriate established category for this tumor, it is classified as NSCLC, NOS, with a distinctive bilayered phenotype. Awareness of this entity is essential to avoid underdiagnosing it as a benign BA/CMPT, and it may eventually be recognized as a distinct tumor type.

## Author Contributions

**Yao Liu:** conceptualization, writing – original draft, data curation, investigation. **Biao Zhang:** methodology, investigation, software. **Xiaobin Cui:** investigation, resources. **Fanqing Meng:** conceptualization, supervision, writing – review and editing.

## Funding

This work was supported by the 2025 Zijing Talent Program—High‐Level Innovative Talent Project.

## Ethics Statement

The authors have nothing to report.

## Consent

Written informed consent for diagnostic procedures and the use of anonymized clinical and pathological data for research and publication was obtained from the patient, as documented in the institutional pathology consent form.

## Conflicts of Interest

The authors declare no conflicts of interest.

## Supporting information


**Supplementary Table 1** Genes included in the SimcereDx Lung cancer targeted therapy comprehensive panel.

## Data Availability

The data that support the findings of this study are available on request from the corresponding author. The data are not publicly available due to privacy or ethical restrictions.
