# Supplementary material for: Non‐Small Cell Lung Carcinoma With Bilayered Architecture and TP53 Alteration: A Diagnostic Pitfall and a Potential Distinct Entity
Source: Thorac Cancer. 2026 Oct 29;17(21):e70411. doi: 10.1111/1759-7714.70411 (PMC13720527; doi:10.1111/1759-7714.70411)
Supplement: Supplementary file 1 [file TCA-17-e70411-s001.doc]

**Supplementary Table 1. Genes included in the SimcereDx Lung Cancer Targeted Therapy Comprehensive Panel.**

| AKT1 | ALK | APC | AR | ARAF | ATM | BCL2L11 | BRAF |
| --- | --- | --- | --- | --- | --- | --- | --- |
| BRCA1 | BRCA2 | CD274 | CDK4 | CDK6 | CDKN2A | CDKN2B | CTNNB1 |
| DDR2 | EGFR | EPCAM | ERBB2 | ERBB3 | ERBB4 | ESR1 | EWSR1 |
| FBXW7 | FGFR1 | FGFR2 | FGFR3 | FLT3 | GNA11 | GNAQ | HRAS |
| IDH1 | IDH2 | JAK1 | JAK2 | KDR | KIT | KRAS | MAP2K1 |
| MDM2 | MET | MLH1 | MPL | MSH2 | MSH6 | MTOR | NF1 |
| NOTCH1 | NRAS | NRG1 | NTRK1 | NTRK2 | NTRK3 | PDCD1 | PDGFRA |
| PDGFRB | PIK3CA | PMS2 | POLE | PTEN | RAF1 | RB1 | RET |
| RICTOR | ROS1 | SDHA | SDHB | SDHC | SDHD | SMAD4 | SMO |
| TP53 | TSC2 | ABCB1 | CBR3 | CDA | CYP2C19 | CYP2C8 | DHFR |
| DYNC2H1 | ERCC1 | GGH | GSTP1 | MTHFR | NQO1 | SOD2 | TYMS |
| UGT1A1 | XPC | XRCC1 |  |  |  |  |  |
